# Dispensary Abuse

**Published:** 1913-12

**Authors:** 


### Dispensary Abuse

The recurrence in a recent issue of the *Buffalo Courier* of a description of the hanging of the three Thayers, which was one of the gala events of early Buffalo history, reminds us that the Dispensary problem has not been discussed in this JOURNAL for a long time, and that the subject has been pretty thoroughly neglected in medical literature lately. The only thing at all new on the subject that occurs to us is, that with the present scale of wages, idleness, intemperance, shiftlessness and a prolonged idleness are the only causes that should justify application for dispensary relief or for any kind of medical charity. This statement needs one further qualification. In a considerable experience in dispensary and municipal relief work, we encountered very few cases that were deserving, taking the normal family as a unit, but the majority were deserving, taking the individual as a unit. That is to say, the majority of cases needed medical relief and were unable to pay for it, but they occurred in little children, in women whose husbands were drunkards or idlers, or in families deprived of the normal supporting head of the family.

What ought to be done in regard to dispensary abuse needs no further discussion. The pertinent question, on which we are unable to speak personally, from recent experience, is whether dispensary abuse exists in western New York, and, if it does exist, what pressure shall be applied to remedy the evil.

